# Pyruvate kinase M2 (PKM2) expression correlates with prognosis in solid cancers: a meta-analysis

**DOI:** 10.18632/oncotarget.13703

**Published:** 2016-11-29

**Authors:** Haiyan Zhu, Hui Luo, Xuejie Zhu, Xiaoli Hu, Lihong Zheng, Xueqiong Zhu

**Affiliations:** ^1^ Department of Obstetrics and Gynecology, The Second Affiliated Hospital of Wenzhou Medical University, Wenzhou 325027, China; ^2^ Department of Gynecology, The First Affiliated Hospital of Wenzhou Medical University, Wenzhou 325027, China

**Keywords:** PKM2, cancer, prognosis, meta-analysis

## Abstract

Pyruvate kinase M2 (PKM2) is the key enzyme in the Warburg effect and plays a central role in cancer cell metabolic reprogramming. Recently, quite a few studies have investigated the correlation between PKM2 expression and prognosis in multiple cancer patients, but results were inconsistent. We therefore performed a meta-analysis to explore the prognostic value of PKM2 expression in patients with solid cancer. Here twenty-seven individual studies from 25 publications with a total of 4796 cases were included to explore the association between PKM2 and overall survival (OS) or disease-free survival (DFS)/ progression-free survival (PFS)/ recurrent-free survival (RFS) in subjects with solid cancer. Pooled analysis showed that high levels of PKM2 was significantly associated with a poorer overall survival (HR = 1.73; 95%CI = 1.48-2.03) and DFS/ PFS/ RFS (HR = 1.90; 95%CI = 1.39-2.59) irrespective of cancer types. Different analysis models (univariate or multivariate models), sample-sizes (≤100 or >100), and methods for data collection (direct extraction or indirect extraction) had no impact on the negative prognostic effect of PKM2 over-expression. Nevertheless, stratified by cancer type, high-expression of PKM2 was associated with an unfavorable OS in breast cancer, esophageal squamous carcinoma, hepatocellular carcinoma and gallbladder cancer; whereas was not correlated with a worse OS in pancreatic cancer and gastric cancer. In conclusion, over-expression of PKM2 is associated with poor prognosis in most solid cancers and it might be a potentially useful biomarker for predicting cancer prognosis in future clinical applications.

## INTRODUCTION

As it is known that cancer has been a major cause of death in both developed and underdeveloped countries; the jeopardy is estimated to grow worldwide due to the increase and aging of the population, as well as a growing prevalence of established risk factors such as smoking, obesity, lack of exercise, and so on [1]. According to GLOBOCAN estimates, approximately 14.1 million new cancer cases and 8.2 million deaths occurred in 2012 worldwide [1]. Combination of surgery, radiotherapy and chemotherapy remain a standard treatment in most cancer cases; however, not all patients derive benefit from these treatment strategies. Therefore, it is of great clinical value to identify a new prognostic marker and personalize treatments according to the individual biology of each cancer.

Pyruvate kinase M2 (PKM2) is a rate-limiting glycolytic enzyme that catalyzes the conversion of phosphoenolpyruvate (PEP) and adenosine diphosphate (ADP) to pyruvate and adenosine triphosphate (ATP) [2, 3]. It has been demonstrated to play a leading role in cancer metabolism and explain the Warburg effect, in which most cancer cells rely on aerobic glycolysis to generate the energy required for cellular processes [4]. Recently, accumulating evidence has suggested that PKM2 is more than a regulator of metabolic reprogramming, suggestive of multiple non-metabolic functions during carcinogenesis. Enhanced expression of PKM2 has been reported in multiple cancers including gastric cancers [5, 6], hepatocellular carcinoma [7], esophageal squamous cell carcinoma [8, 9], colorectal cancer [10], and gallbladder cancer [11]. In addition, more recently, quite a few studies have investigated the correlation between the expression of PKM2 and prognosis among multiple cancer patients. An overwhelming majority of evidence has explored an unfavorable prognostic value of PKM2 over-expression in a wide spectrum of cancers [8, 11–13], however, due to variance in tumor type, study design and sample size, several studies failed to draw similar conclusions. Lockney et al. [14] reported that positive PKM2 expression predicted improved overall survival in pancreatic ductal adenocarcinoma patients. Benesch et al. [15] reported that strong PKM2 expression indicated a favorable outcome for breast cancer patients.

Therefore, it is necessary to systematically clarify the prognostic significance of PKM2 in cancers through meta-analysis of current data. Our study was designed to assess the correlation between PKM2 expression and overall survival (OS) as well as disease-free survival (DFS)/ progression-free survival (PFS)/ recurrence-free survival (RFS) in solid cancer patients by pooling results from published data. Thereby, we intend to shed more light on the clinical value of PKM2 as a prognostic indicator and as a target for therapeutic intervention. To the best of our knowledge, this is the first meta-analysis to comprehensively investigate the prognostic value of PKM2 expression among solid cancer patients.

## RESULTS

### Literature search results

**Figure 1:**
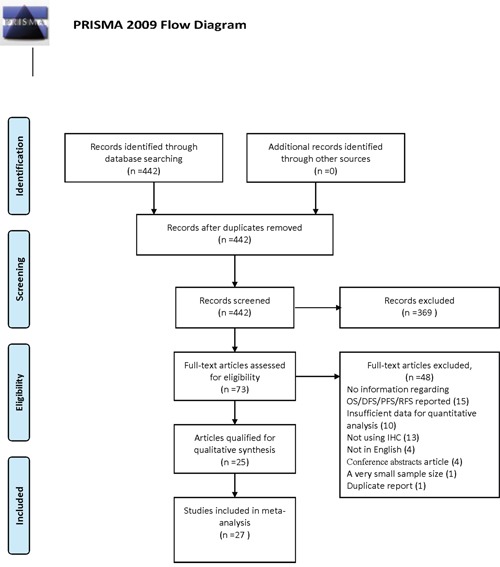
Flow chart of study selection in the meta-analysis

Our search yielded 442 records; of them, 369 were excluded as irrelevant on the basis of title and abstract. Further assessment for more detailed information identified 73 articles, of which 48 publications were excluded (15 have no information regarding OS/DFS/PFS/RFS; 10 had insufficient data for quantitative analysis; 1 had a very small sample size (n<30) [16] ; 4 not in English; 4 conference abstracts; 13 measured the expression of PKM2 not by immunohistochemistry (IHC), 1 duplicate report). Details of the study selection process are shown in Figure 1. Among the remaining 25 articles, two separate studies both analyzed two distinct cohorts [11, 17]. Data from the individual cohorts was extracted as two individual studies, resulting in 4 studies from 2 publications. Thus, twenty-seven individual studies from 25 publications with a sum of 4796 cases were involved in this meta-analysis.

### Characteristics of included studies

The basic characteristic descriptions of the 27 eligible studies are summarized in Table 1 [5–14, 17–31]. These studies were conducted in 6 countries (24 cohorts were Asian populations and 3 cohorts were Caucasian populations), and they were published between 2012 and 2016. The mean sample size was 178 patients (ranged, 36-484). Studies concerning hepatocellular carcinoma (n=5) and pancreatic cancer (n=5) occupied the largest proportion of cancer type among all primary literatures, followed by esophageal squamous cell carcinoma (n = 4), then gastric cancer (n=3), breast cancer (n = 2), gallbladder cancer (n=2), and remaining types of solid cancers (n=1). A total of 24 studies described the correlation between overall survival and PKM2 expression, while 9 datasets reported a relationship between DFS/ PFS/ RFS and PKM2 expression. All of the eligible entries scored more than five by Newcastle-Ottawa Scale, revealing a high methodological quality across all studies.

**Table 1:** Characteristics of eligible studies

Study & year	Country	Ethnicity	Cancer type	Sample size (n)	Age Medium (Min-Max)	Gender (Female/male)	PKM2 (high/low) No.	Follow-up (months) Medium(Min-Max)	Outcomes	HR (95%CI)	Method for data collection	Model	NOS score
Calabretta 2015[15]	Italy	Caucasian	Pancreatic cancer	42	NA	20/22	26/16	NA	PFS	1.12 (1-4.4)	Directly	M	6
Chen 2015[5]	China	Asian	Hepatocellular carcinoma	205	NA	33/172	138/67	29 (1-141)	OS	1.72 (1.14-2.59)	Directly	M	8
Cui 2015[8]	China	Asian	Colorectal cancer	183	NA	NA	136/47	NA	OS	3.71 (2.23-6.17)	Directly	M	7
Dong 2015[16]	China	Asian	Breast cancer	295	50 (24-76)	All female	135/160	72.2 (5-134)	OS	1.84 (1.4-2.42)	Directly	U	7
Dong 2015[16]	China	Asian	Breast cancer	295	50 (24-76)	All female	135/160	61.6 (1-134)	PFS	2.65 (1.87-3.27)	Directly	U	7
Fukuda 2015[10]	Japan	Asian	Esophageal carcinoma	205	NA	30/175	104/101	47.9± 43.4	OS	1.85 (1.2-2.78)	Directly	M	7
Gao 2015[17]	China	Asian	Gastric cancer	124	59.5	40/84	47/77	38 (1-108)	OS	1.23 (0.67-2.25)	Directly	M	8
Hu 2015[18]	China	Asian	Hepatocellular carcinoma	484	49 (13-68)	NA	252/232	25.9	OS	1.52 (1.28-1.8)	Directly	M	7
Hu 2015[18]	China	Asian	Hepatocellular carcinoma	411	49 (13-68)	NA	206/205	25.9	PFS	1.64 (1.3-2.07)	Directly	M	7
Kwon 2012[4]	Korea	Asian	Gastric cancer	380	NA	NA	159/221	NA	OS	1.56 (1.04-2.34)	Indirectly	U	6
Li 2014[19]	China	Asian	Esophageal carcinoma	141	60	54/87	59/82	NA	OS	1.21 (0.73-2.03)	Directly	M	6
Li 2014 a[9]	China	Asian	Gallbladder cancer	80	NA	54/26	45/35	2 years	OS	2.17 (1.39-3.39)	Indirectly	U	7
Li 2014 b[9]	China	Asian	Gallbladder cancer	46	NA	27/19	26/20	2 years	OS	1.96 (1-3.85)	Indirectly	U	7
Li 2016[20]	China	Asian	Pancreatic cancer	90	NA	33/57	65/25	NA	OS	2.41 (1.08-5.42)	Indirectly	U	6
Lim 2012[3]	South Korea	Asian	Gastric cancer	368	NA	146/222	144/224	70.6 (3.6-144.6)	OS	0.92 (0.65-1.3)	Directly	U	8
Lim 2012[3]	South Korea	Asian	Gastric cancer	368	NA	146/222	144/224	70.6 (3.6-144.6)	PFS	0.93 (0.66-1.32)	Directly	U	8
Lin 2015[11]	China	Asian	Breast cancer	296	51 (23-83)	All female	153/143	52 (2-82)	OS	1.9 (0.98-3.71)	Directly	M	6
Lin 2015[11]	China	Asian	Breast cancer	296	51 (23-83)	All female	153/143	52 (2-82)	PFS	2.14 (1.16-3.93)	Directly	M	6
Liu 2015a[14]	China	Asian	Hepatocellular carcinoma	367	NA	48/319	89/278	52.2	OS	1.9 (1.32-2.74)	Directly	M	8
Liu 2015b[14]	China	Asian	Hepatocellular carcinoma	354	NA	55/299	70/284	52.2	OS	1.57 (1.05-2.35)	Directly	M	8
Lockney 2015[12]	USA	Caucasian	Pancreatic cancer	115	67 (57-73)	52/63	61/54	NA	OS	0.57 (0.36-0.91)	Directly	M	7
Mohammad 2016[21]	UK	Caucasian	Pancreatic cancer	72	NA	33/39	46/26	NA	OS	1.68 (1.05-2.67)	Indirectly	U	6
Ogawa 2015[22]	Japan	Asian	Pancreatic cancer	36	70 (47-83)	15/21	16/20	NA	OS	2.16 (0.82-6.1)	Directly	M	6
Sun 2015[23]	China	Asian	Lung adeno- carcinoma	65	60 (28-75)	20/45	28/37	NA	RFS	1.89 (0.73-4.9)	Indirectly	U	7
Wang 2015[24]	China	Asian	Oral carcinoma	111	52.8	61/60	63/48	51.4 (3-78)	OS	3.12 (1.45-5.08)	Directly	M	7
Wang 2015[24]	China	Asian	Oral carcinoma	111	52.8	61/60	63/48	51.4 (3-78)	PFS	2.36 (1.03-5.44)	Indirectly	U	7
Wong 2014[25]	China	Asian	Hepatocellular carcinoma	109	NA	NA	75/34	NA	OS	1.69 (1.7-4.05)	Indirectly	U	6
Yu 2015[26]	China	Asian	Hilar cholangio- carcinoma	88	55 (31-79)	26/62	47/41	16 (1-59)	DFS	3.2 (1.75-5.84)	Indirectly	U	7
Yu 2015[26]	China	Asian	Hilar cholangio-carcinoma	88	55 (31-79)	26/62	47/41	16 (1-59)	OS	2.67 (1.39-5.14)	Indirectly	U	7
Yuan 2014[27]	China	Asian	Tongue cancer	63	54.7 (26-74)	26/37	42/21	46.8 (2-80)	OS	6.02 (1.51-23.93)	Directly	M	7
Zhan 2013[7]	China	Asian	Esophageal carcinoma	126	NA	31/95	84/42	NA	OS	2.21 (0.92-5.3)	Indirectly	U	7
Zhang 2013[6]	China	Asian	Esophageal carcinoma	86	65 (41-81)	22/64	61/25	NA	OS	2.36 (1.16-4.81)	Directly	M	6
Zhao 2015[28]	China	Asian	Cervical cancer	132	51 (28-80)	All female	90/42	45 (2-85.5)	PFS	2.89 (1.35-6.19)	Directly	M	7

Abbreviations: HR = hazard ratio; CI = confidence interval; NOS = Newcastle-Ottawa Scale, NA = not available; U = univariate; M = multivariate; OS = overall survival; DFS = disease-free survival; PFS = progression-free survival; RFS = recurrence-free survival.

### Quality assessment of relationship between PKM2 expression and OS

**Figure 2:**
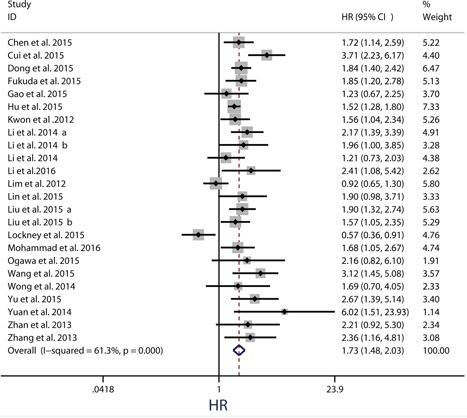
Meta-analysis of impact of PKM2 expression on overall survival of patients with solid cancers Results are presented as individual and pooled HR, and 95% CI.

**Figure 3:**
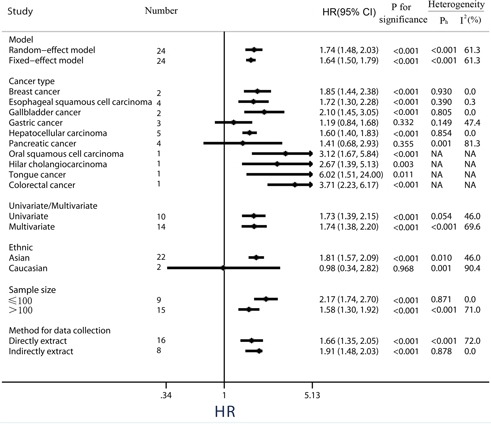
Subgroup analysis of the association between PKM2 expression and overall survival of solid cancers

Twenty-four observational trials offered original data on overall survival in ten types of cancers. The synthesis indicated that over-expression of PKM2 was significantly related to a poorer OS (pooled HR = 1.73, 95%CI = 1.48-2.03) (Figure 2), and these associations were demonstrated both in univariate models (pooled HR = 1.73, 95%CI = 1.39–2.15) and multivariate models (pooled HR = 1.74, 95%CI = 1.38–2.20) (Figure 3). Because moderate heterogeneity was observed (*P* = 0.000, *I^2^* = 61.3%), we utilized a random-effects model to determine the pooled HR and 95% CI. Moreover, subgroup meta-analysis was performed to investigate the possible source of the heterogeneity among studies, according to various confounding factors (Figure 3).

In the stratified analysis by tumor type, high levels of PKM2 were significantly correlated with a poorer OS for patients with breast cancer (pooled HR = 1.85, 95%CI = 1.44-2.38), esophageal squamous cell cancer (pooled HR = 1.72, 95%CI = 1.30-2.28), gallbladder cancer (pooled HR = 2.10, 95%CI = 1.45-3.05) and hepatocellular carcinoma (pooled HR = 1.60, 95%CI = 1.40-1.83). Nevertheless, over-expression of PKM2 in gastric cancer (3 studies, pooled HR = 1.19, 95%CI = 0.84-1.68, *P* = 0.332, *I^2^* = 47.4%) and pancreatic cancer (4 studies, pooled HR = 1.41, 95%CI = 0.68-2.93, *P* = 0.355, *I^2^* = 81.3%) has no effect on OS, along with a moderate heterogeneity observed in pancreatic cancer (Figure 3).

Subgroup analyses by ethnicity revealed that PKM2 was an unfavorable predictor of OS in Asian populations (pooled HR = 1.81, 95%CI = 1.57-2.09), whereas PKM2 expression implied a better outcome trend in Caucasian populations along with a significant heterogeneity and a *P* value of more than 0.05 (pooled HR = 0.98, 95% CI: 0.34-2.82, *P* = 0.968, *I^2^* = 90.4%).

Because some individual HRs were indirectly estimated (see Materials and Methods) and were therefore less reliable, we also performed subgroup analyses according to this method of data collection. Results showed high levels PKM2 predicted an unfavorable OS among studies data collection directly (pooled HR = 1.66, 95%CI = 1.35-2.05), as well as those data collection indirectly (pooled HR = 1.91, 95%CI = 1.48-2.03) (Figure 3). Among the subgroup divided by different amount of sample size, either small sample size study (sample sizes ≤100, pooled HR = 2.17, 95%CI = 1.74-2.70), or large sample size study (sample sizes > 100, pooled HR = 1.58, 95%CI = 1.30-1.92), the merged outcome consistently indicated a worse OS among patients with PKM2 over-expression.

### Quality assessment of relationship between PKM2 expression and DFS/PFS/RFS

**Figure 4:**
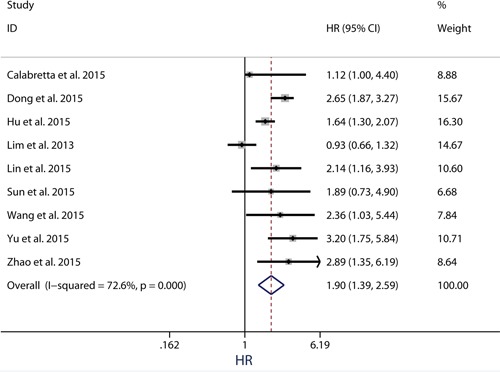
Meta-analysis of impact of PKM2 expression on disease-free survival/progression-free survival/recurrence-free survival of patients with solid cancers Results are presented as individual and pooled HR, and 95% CI.

**Figure 5:**
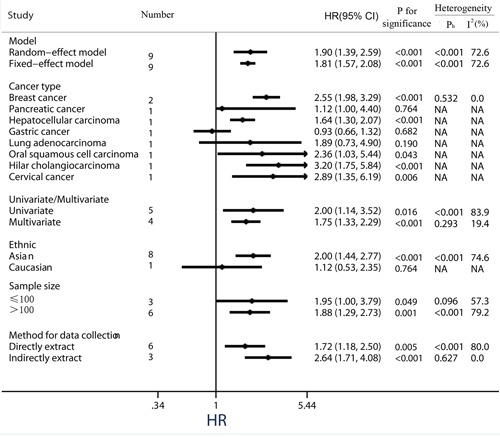
Subgroup analysis of the association between PKM2 expression and disease-free survival/ progression-free survival/ recurrence-free survival of solid cancers

Because the outcome endpoints DFS, PFS and RFS are similar in meaning, they were combined to make a unified prognostic parameter, time to tumor progression (TTP) was used for the meta-analysis [32]. Meta-analysis of DFS/PFS/RFS was conducted in 9 studies (Figure 4). The pooling analysis revealed high-expression of PKM2 was a negative indicator for DFS/PFS/RFS among solid cancer patients, with a pooled HR 1.90 (95%CI = 1.39-2.59) in random model and a pooled HR 1.81 (95%CI = 1.57-2.08) in fixed model. Because the heterogeneity test reported a *P* value of less than 0.01, the random-effect model was used to determine the summary of DFS/PFS/RFS. This association was noteworthy not only in univariate models (pooled HR = 2.00, 95%CI = 1.14-3.52), but also in multivariate models (pooled HR = 1.75, 95%CI = 1.33-2.29), suggestive of a noteworthy relationship between high levels of PKM2 and unfavorable clinical outcome (Figure 5).

When considering differences in ethnicity, high PKM2 expression status was identified as a worse prognostic marker of time to tumor progression in the Asian group (pooled HR = 2.00, 95%CI = 1.44-2.77; *P* <0.001). Nevertheless, there was no significant correlation between PKM2 over-expression and shorter DFS/PFS/RFS among patients in the Caucasian group (pooled HR = 1.12, 95%CI = 0.53-2.35; *P* = 0.764) (Figure 5). In addition, subgroup analyses showed that elevated PKM2 levels predicted the poor prognosis for solid cancer patients, regardless of data collection methods (direct extraction or indirect extraction) or sample sizes (sample sizes > 100 or sample sizes ≤ 100).

### Publication bias

**Figure 6:**
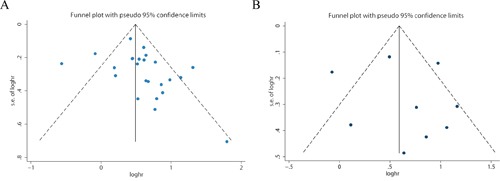
Funnel plot for the assessment of potential publication bias in the impact of PKM2 on overall survival **A.** disease-free survival/ progression-free survival/recurrence-free survival **B.**

Publication bias was investigated by the funnel plots, Begg's and Egger's tests. Visual inspection of the funnel plots did not show obvious asymmetry for OS (Figure 6A), or DFS/RFS/PFS (Figure 6B) analyses. Additionally, Egger's test (*P* = 0.121 for OS; *P* = 0.686 for DFS/RFS/PFS) and Begg's test (*P* = 0.107 for OS; *P* = 0.917 for DFS/RFS/PFS) further confirmed that there was no publication bias among the included studies in this meta-analysis.

### Sensitivity analysis

**Figure 7:**
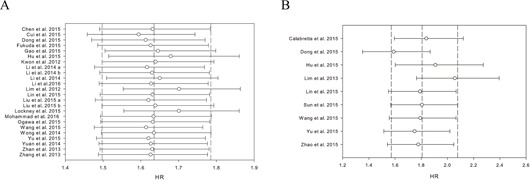
Results of leave-one-out method in the impact of PKM2 on overall survival **A.** disease-free survival/ progression-free survival/ recurrence-free survival **B.**

Sensitivity analyses were used to evaluate whether individual studies influenced the results. The leave-one-out method, i.e. leaving out one study in turn to explore the stability of the obtained conclusions, was adopted. As shown in Figure 7, the statistical significance of the results was not changed when any single study was omitted. This observation further confirmed the stability of the results. Thus, the results of this meta-analysis are stable and robust.

## DISCUSSION

PKM2 is an important cancer metabolism enzyme responsible for the Warburg effect. In highly glycolytic cancers, the conversion of phosphoenolpyruvate (PEP) and adenosine diphosphate (ADP) to pyruvate and adenosine triphosphate (ATP) in the presence of oxygen (‘aerobic glycolysis’) generates the necessary amount of energy needed for rapid cellular proliferation [4]. Indeed, enhanced expression of PKM2 is frequently observed in various human cancers and is important for tumor initiation, progression and chemoresistance [33]. Individual cohorts have sporadically revealed an unfavorable impact of high PKM2 expression on clinical prognosis in certain types of cancer. However, it still remains unclear if PKM2 expression can consistently predict outcomes in patients with solid cancers among a wide range of tumor grades and types.

Here twenty-seven individual studies from 25 publications with a sum of 4796 cases were included to evaluate the prognostic value of PKM2 in subjects with solid cancer. The results of our meta-analysis suggest that over-expression of PKM2, which indicates a higher rate of glycolysis in tumor cells, is associated with an unfavorable prognosis and is a potential biomarker associated with OS and DFS/PFS/RFS in patients with solid tumors.

Regarding the tumor types, elevated PKM2 expression in tumor tissues predicted a worse OS in individuals with breast cancer, esophageal squamous cell cancer, gallbladder cancer and hepatocellular carcinoma. However, the correlation between PKM2 over-expression and OS was inconclusive in gastric cancer and pancreatic cancer. Further studies are required to clarify the role of PKM2 as a biomarker for prognosis in these types of cancers.

With respect to ethnicity/race, increased levels of PKM2 had a negative influence on clinical outcome in the Asian group, with consistent results of OS and DFS/PFS/RFS. Nevertheless, in the Caucasian population, PKM2 over-expression implied a favorable overall survival trend (*P* = 0.968) and showed no significant correlation with DFS/PFS/RFS. Although quite a few genes exert different effects on cancer risk and prognosis across ethnic groups, Liu et al. [34] analyzed differentially-expressed proteins in esophageal cancer between three ethnic groups in Xinjiang Kazakh, Uygur and Han's by protein profiles and reported PKM2 expression showed no difference in cancer risk or prognosis among these three ethnic groups. Given that only three studies containing Caucasian patients were included in this meta-analysis, we believe these differences were due to the small sizes of available studies and different cancer types, rather than specific ethnics. Therefore, further investigations are needed to clarify the role of PKM2 as a biomarker for prognosis in some types of cancer, especially in Caucasian populations.

Although PKM2 has been investigated for its role in cancer for almost 40 years, the underlying mechanisms involved in the association between over-expression of PKM2 and decreased OS remains elusive. One possible explanation is its capability of promoting the progression of human cancers [21]. For instance, PKM2 has been demonstrated to promote cell proliferation, migration, resistance to apoptosis, angiogenesis, autophagy, intratumoral inflammatory cell infiltration, and pre-metastatic niche formation in hepatocellular carcinomas. [17] PKM2 also stimulates glycolysis and lipid synthesis, thereby promoting cell proliferation and invasion in lung adenocarcinomas [26]. Kwon et al. [6] reported that PKM2 affected gastric cancer cell survival by regulating Bcl-xL at the transcriptional level, elucidating a potential explanation as to why high levels of PKM2 relate to an unfavorable clinical outcome. In further support, Hu et al. [21] showed that PKM2 depletion can result in cell apoptosis induced by stabilization of the proapoptotic protein Bim.

Another feasible explanation involves PKM2 expression and its association with chemo-resistance [12] and radiation resistance [31]. To be more specific, PKM2 expression was related to poor response to chemotherapy in esophageal squamous cell carcinoma patients [12] as well as radiation resistance [31] in cervical cancer patients.

This study has several important implications. First, it shows that PKM2 expression is related to worse outcome of solid cancers, which suggests that PKM2 may be a potential prognostic indicator for solid cancer. Second, this study restricts PKM2 detection methods to immunohistochemistry staining, which is the primary technique used to determine protein expression status in patient samples. These techniques have been widely used in the morphological diagnosis of malignancy, determining the primary site of tumor origin, and benefiting the treatment decisions and prognosis. Its major advantage pertains to specimen acquisition. Thus, it will not only decrease the heterogeneity, but can also be easily translated into clinical applications. Third, all of the analyses were conducted by random-effects and fixed effects models. Both models showed similar results, which indicated that the statistic results were stable and robust. Finally, this study emphasizes the importance of developing a valuable biomarker for prognostic assessment of solid cancers.

Some limitations also exist in this meta-analysis. First, from the literature we could only extract summarized population-level data rather than individual patient-level data. In addition, the HR of some studies was estimated indirectly as previously reported [35, 36]. These data were less reliable compared to direct data from the original literature. Second, moderate heterogeneity observed across studies due to confounding factors such as the clinical features of the patients, ethnicity, sample size, HR estimation and PKM2 cut-off value, which cannot be completely accounted for in spite of using suitable meta-analytic techniques and subgroup analyses. As an example, the vast majority of included publications employed samples of Asian ethnicity, thus the evaluation of outcome in Caucasians might be derived by chance because of sample insufficiency. Finally, studies with small sample size and negative results may not be published, which can cause publication bias [37], potentially overstating the correlation between PKM2 expression and unfavorable clinical outcomes. Therefore, further investigations are needed to address the above-mentioned shortcomings.

## MATERIALS AND METHODS

We performed this meta-analysis in accordance with the Preferred Reporting Items for Systematic Reviews and Meta-Analyses (PRISMA) statement [38].

### Search strategy and selection criteria

In accordance with the PRISMA guidelines, we identified studies through a systematic review of Medline (via PubMed), Cochrane database, and EMBASE (via Ovid) from the inception to Mar 21, 2016, using the following search terms: (PKM2 OR M2 pyruvate kinase isoenzyme OR M2 isoform of pyruvate kinase OR pyruvate kinase isoform M2 OR pyruvate kinase M2) AND (cancer OR carcinoma OR neoplasm OR malignancy OR tumor). We also checked reference lists and citation histories during the search. To ensure the quality of the meta-analysis, two authors (Haiyan Zhu & Hui Luo) independently performed the search and identification according to the standardized approach, and the final selection of a study for inclusion in the meta-analysis was reached in consensus. The following inclusion criteria were used in the meta-analysis: (1) the publication explored the relation between PKM2 expression and solid tumor prognoses, such as OS, DFS, PFS and RFS; (2) the expression of PKM2 was detected in tumor tissue, rather than in the serum or cell lines or any other kinds of specimens; (3) they measured the expression of PKM2 by the standard methods of immunohistochemistry and reported the corresponding cut-off value; (4) there were sufficient, clear, and available data to extract or estimate HR and 95% CI; (5) each study had a size of greater than thirty individuals; (6) studies were published in English; (7) the meta-analysis was restricted to original articles (no expert opinions, editorials or reviews). Conference abstracts and other unpublished articles were also excluded. Studies were excluded if they did not meet all criteria. If one study reported multi-datasets based on different populations, datasets would be recognized individually. For multiple publications reporting the same study, only the most informative or most recent publication was included in the meta-analysis. The approval of the study was obtained from the local research ethics committee.

### Data extraction and quality assessment

All eligible publications were reviewed independently by two investigators (HYZ, HL), who both extracted the data using predefined data abstraction forms. Disagreements were resolved by discussion. For each eligible study, the following data was extracted: first author's name, year of publication, country of origin, type of cancer, number of patients, median age, gender, number of PKM2 over-expression patients and controls, tumor stage, median and range of follow-up time, outcome endpoint, univariate or multivariate HR and 95% CI for PKM2 over-expression (exposed group) versus PKM2 low-expression (unexposed group). If the studies showed inadequate or unclear information, sending an email to the authors for complementary information was our first choice. If the Kaplan-Meier survival curves were available, we used the method previously described by Greenland et al. [36] and Tierney et al. [35] to estimate HR and its corresponding 95% CI. Multivariate HR and 95% CI were selected if both univariate and multivariate results were reported in an individual study.

Based on the extracted data, the quality of the included studies was evaluated by the Newcastle-Ottawa Scale (NOS), a widely used tool for the quality assessment of observational or non-randomized studies [39]. Using this ‘star system’, each included study was judged on three broad perspectives: the selection of the study groups; the comparability of the groups; and the ascertainment of outcome of interest. Studies scoring 6 or higher were classified as high-quality studies. A consensus NOS score for each item was achieved.

### Statistical analysis

The effect of PKM2 over-expression on the outcomes of solid cancer patients was measured by HR with 95% CI, and the HRs from relevant studies were combined to produce a summary HR for each outcome. Outcome endpoints were divided into two groups, OS and DFS/RFS/PFS, based on the data acquired in the current study and previous reports. A combined HR >1 implied a worse prognosis of patients with PKM2 over-expression, while HR <1 means the opposite.

Heterogeneity was quantified and evaluated by the chi-squared-based Q-test and I^2^ test, with *P*<0.05 and *I^2^*>50% indicating evidence of heterogeneity. In case of substantial heterogeneity the random-effects model was used (Der Simonian-Laird method) [40]; otherwise, the fixed-effects model was used (Mantel-Haenszel method) [41]. Potential publication bias was analyzed by performing funnel plots qualitatively, and estimated by Begg's and Egger's test quantitatively [42, 43]. Sensitivity analyses were conducted by omitting one study each time and by using the alternative analysis model (e.g. switching from the random effects model to the fixed-effects model) [44]. All analyses were conducted using Stata 12.0 software (StataCorp LP, College Station, TX). For all analyses, a two-sided *P* value less than 0.05 was considered to be statistically significant.

## CONCLUSIONS

Taken together, this meta analysis demonstrates that over-expression of PKM2 in solid tumor tissues, as measured by immunohistochemistry, is associated with a poor prognosis in most solid tumors, which suggests that PKM2 might be a potential prognostic biomarker and targeting PKM2 could be a promising therapeutic approach for treating solid tumors in a variety of cancer types.
